# Olfactory disorders in coronavirus disease 2019 patients: a systematic literature review

**DOI:** 10.1017/S0022215120002005

**Published:** 2020-09-15

**Authors:** E Fuccillo, A M Saibene, M P Canevini, G Felisati

**Affiliations:** 1 Department of Clinical Sciences and Translational Medicine, ‘Tor Vergata’ University of Rome, Italy; 2 Department of Health Sciences, University of Milan, Italy

**Keywords:** COVID-19, SARS-CoV-2, Smell, Olfactory Nerve Diseases, Olfaction Disorders

## Abstract

**Objective:**

Recent scientific literature has widely described a possible major role of smell dysfunction as a specific symptom of coronavirus disease 2019. This systematic review may provide a more holistic approach to current knowledge of the disease.

**Methods:**

A systematic review was completed using Embase, PubMed and Web of Science databases that considered original articles focused on olfactory evaluation in coronavirus disease 2019 patients, published between March and May 2020, in English language.

**Results:**

From the 483 research papers initially identified, 32 original studies were selected, comprising a total of 17 306 subjects with a laboratory confirmed diagnosis of coronavirus disease 2019. Individual study sample sizes ranged from 6 to 6452 patients. This comprehensive analysis confirmed that olfactory disorders represent an important clinical feature in coronavirus disease 2019, with a prevalence of 11–100 per cent in included patients, although there was heterogeneity in terms of assessment tools and population selection criteria.

**Conclusion:**

The results indicate that an accurate clinical evaluation should be carried out using structured questionnaires and tests with olfactory substances.

## Introduction

Infection by the new pathogen severe acute respiratory syndrome coronavirus-2 (SARS-CoV-2) has highlighted a possible major role of chemosensory dysfunction, with a particular reference to smell disorders, often in association with taste disorders.^1,2^

Focusing on smell impairment, it is known that post-viral anosmia could be a fairly common sequela of upper airway disease.^3^ However, the clinical presentation of smell disorders during coronavirus disease 2019 (Covid-19) does not seem to be ‘univocal’, ranging from patient reports of normal smell, to reports of partial loss of smell (hyposmia) or total loss of smell (anosmia), or even altered perception of smell (dysosmia).

Many research teams have evaluated olfactory dysfunction in patients affected by Covid-19, highlighting a possible role of the viral invasion of the olfactory bulb by SARS-CoV-2 as the main aetiopathogenic mechanism of olfactory dysfunction.^4^ Bulfamante *et al*. recently described the autoptic presence of numerous particles, likely referable to virions of SARS-CoV-2, at the level of the olfactory nerve.^5^

However, it remains difficult to establish the exact prevalence of smell disorders, the expected timing of onset, the smell outcome, the associated risk factors, the relationship with taste disorders and, above all, the aetiopathogenetic mechanisms of damage.

A small number of systematic reviews^6–12^ have been published already, during the early stages of the pandemic in Europe and USA. However, in light of continuous scientific updating, we believe that our study can provide a more holistic approach to current knowledge of the disease. Furthermore, we believe that accurate identification of an olfactory disorder and its characteristics could facilitate our understanding of pathogenetic mechanisms, with particular reference to possible involvement of the central nervous system, thus ultimately enhancing our wider understanding of the role of smell dysfunction in Covid-19.

## Materials and methods

**Fig. 1.**
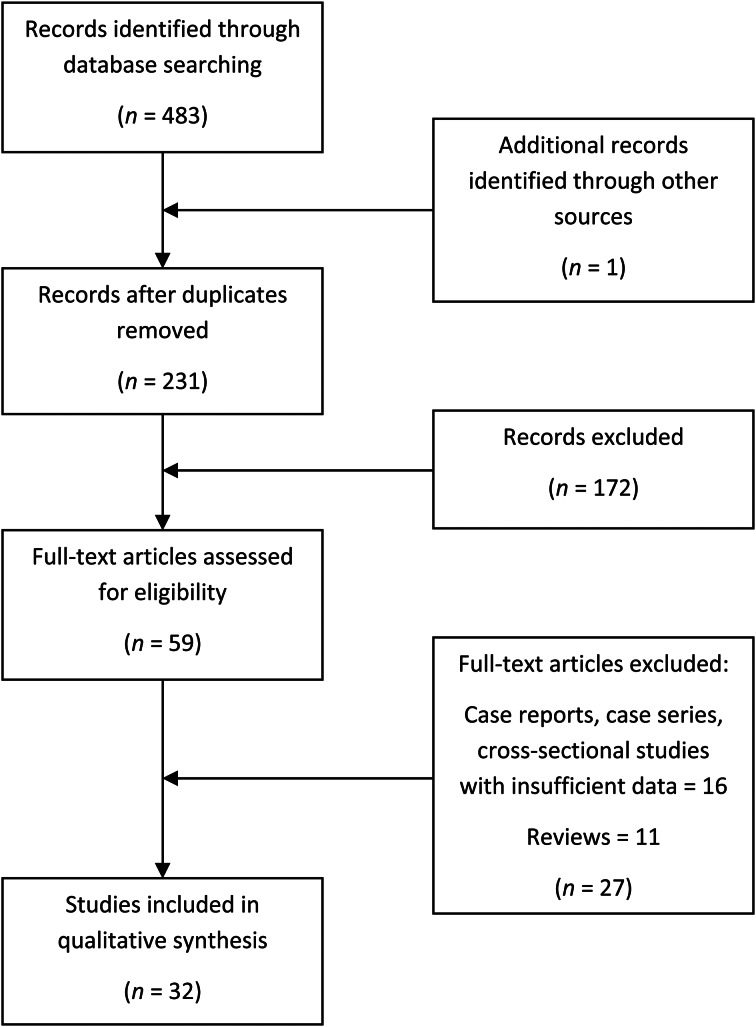
Preferred Reporting Items for Systematic Reviews and Meta-Analyses (‘PRISMA’) flowchart.

This research was conducted using PubMed, Embase and Web of Science databases (Table 1), focusing on papers published up to 31th May 2020. The search was carried out according to the Preferred Reporting Items for Systematic Reviews and Meta-Analyses (‘PRISMA’) reporting guidelines,^13^ as shown in Figure 1. Specifically, we performed a systematic electronic search of original articles published between March and May 2020, in English language, considering studies focused on olfactory evaluation in Covid-19 patients. Although there are many studies that consider smell dysfunction in patients affected by Covid-19, we chose to specifically consider only those which carried out an in-depth assessment focused on chemosensory disorders, particularly smell impairment, in Covid-19 patients.

**Table 1.** Summary of search strategies

Database	Search strategy	Date of search	Unique papers found (*n*)
PubMed	((“COVID” OR “COVID-19” OR “SARS-COV-2” OR “coronavirus”)) AND (“smell” or “anosmia” or “dysosmia” or “hyposmia” or “parosmia” or “olfaction” or “olfactory”)	31 May 2020	199
Embase	(‘coronavirus’ OR ‘covid’ OR ‘covid 19’ OR ‘sars cov 2’) AND (‘smell’ OR ‘anosmia’ OR ‘dysosmia’ OR ‘hyposmia’ OR ‘parosmia’ OR ‘olfaction’ OR ‘olfactory’)	31 May 2020	216
Web of Science	(TS= (Covid 19 OR Covid OR Coronavirus OR SARS-COV-2)) AND (TS= (Smell OR anosmia OR dysosmia OR hyposmia OR parosmia OR olfaction OR olfactory))	31 May 2020	68

Inclusion criteria were: a laboratory confirmed diagnosis of Covid-19 infection; and the presence of a smell evaluation assessed through anamnestic and/or database data collection, a simple survey, a validated questionnaire focused on olfactory ability, and/or chemosensitive tests with odorants. We excluded from our investigation all systematic and narrative reviews, case reports, and all studies without specific data on patients affected by Covid-19. For a more precise analysis, we also excluded studies in which the patient's setting and/or smell evaluation method was not clearly explained.

The references of review articles were checked for cross-referencing purposes. The research process was conducted by two different authors (EF and AMS). Disagreements regarding the final selection of studies were discussed by the two authors and a final consensus was reached.

For each included article, we recorded: the number of Covid-19 patients, the number of patients with olfactory dysfunction, the country (and city if available) in which the study was performed, the type of study, patients’ data, the adopted method for smell evaluation (anamnestic data collection, simple survey, elaborated questionnaire focused on olfactory ability and/or chemosensitive tests with odorant), the time of evaluation, the time of disease onset, the concomitant evaluation of taste disorders, the patient setting (in-patient and/or out-patient) and evaluation results.

The selected studies were assessed for quality and methodological bias using the National Heart, Lung, and Blood Institute Study Quality Assessment Tools.^14^ The level of evidence was assessed according to the Oxford Centre for Evidence-Based Medicine level of evidence guide.^15^

### Patients, intervention, comparison and outcomes criteria

The patients, intervention, comparison and outcomes (‘PICO’) criteria for the review were considered as follows: (1) patients – patients with SARS-CoV-2 infection certified on laboratory tests who underwent a clinical evaluation of smell impairment using anamnestic data, a smell questionnaire and/or olfactory tests; (2) intervention – clinical evaluation of olfactory disorders; (3) comparison – different methods of evaluating olfactory function (subjective and objective); and (4) outcome – prevalence and characteristics of olfactory dysfunction in Covid-19 patients.

## Results

Of the 483 research papers initially identified, 32 original studies were finally selected, comprising a total of 17 306 subjects with a laboratory confirmed diagnosis of Covid-19. Individual study sample sizes ranged from 6 to 6452 patients. The studies’ characteristics are described in Table 2.^2,16–46^ Over half of the selected studies were carried out in European countries.

**Table 2.** Summary of included studies

Study authors	Covid-19 patient population size (*n*)	Patients with olfactory dysfunction	Study location	Study type	Oxford level of evidence	NHI-SQAT score
Aggarwal *et al*.^16^	16	3 (19%) subjective olfactory &/or taste dysfunction	Des Moines, USA	Retrospective cohort study	4	Fair
Beltrán-Corbellini *et al*.^17^	79	25 (31.65%) subjective olfactory dysfunction	Madrid, Spain	Case series	4	Fair
Carignan *et al*.^18^	134	87 (64.9%) subjective olfactory &/or taste dysfunction	Quebec Eastern Townships, Canada	Retrospective cohort study	4	Fair
Giacomelli *et al*.^19^	59	20 (33.9%) subjective olfactory &/or taste dysfunction	Milan, Italy	Cross-sectional study	4	Fair
Hornuss *et al*.^20^	45	38 (84%) objective olfactory dysfunction	Freiburg, Germany	Cross-sectional study	4	Good
Kai Chua *et al*.^21^	31	7 (22.6%) subjective olfactory dysfunction	Singapore	Cross-sectional study	4	Fair
Kim *et al*.^22^	213	68 (31.9%) subjective olfactory dysfunction	Seoul, South Korea	Cross-sectional study	4	Good
Klopfenstein *et al*.^23^	114	54 (47.4%) subjective olfactory dysfunction	Trévenans, France	Retrospective cohort study	4	Fair
Lechien *et al*.^24^	417	357 (85.6%) subjective olfactory dysfunction, with validated tool	12 European hospitals	Cross-sectional study	4	Good
Lechien *et al*.^25^	2013; subset of 93 patients were eligible for objective olfactory evaluation	1754 (87%) subjective olfactory dysfunction	18 European hospitals	Cross-sectional study	4	Good
Lechien *et al*.^26^	86	53 (62%) objective olfactory dysfunction	Mons, Belgium	Cross-sectional study	4	Good
Lechien *et al*.^27^	1420	70.2% subjective olfactory dysfunction	18 European hospitals	Cross-sectional study	4	Fair
Lee *et al*.^28^	3191	488 (15.3%) subjective mixed olfactory &/or taste dysfunction in patients at early stage of Covid-19	Daegu, South Korea	Cross-sectional study	4	Good
Li *et al*.^29^	145	16 (11%) objective olfactory dysfunction 25 days from symptom onset	Wuhan, China	Cross-sectional study	4	
Luers *et al*.^30^	72	53 (73.61%) subjective dysfunction	Cologne, Germany	Retrospective cohort study	4	Fair
Menni *et al*.^31^	6452 in UK, 726 in USA	64.8% in UK & 67.5% in USA had subjective olfactory &/or taste dysfunction	UK & USA	Cross-sectional study	4	Good
Moein *et al*.^32^	60	59 (98.33%) objective olfactory dysfunction, 21 (35%) subjective olfactory &/or taste dysfunction	Teheran, Iran	Case series	4	Fair
Noh *et al*.^33^	199	52 (26.1%) subjective olfactory dysfunction	Gyeongju, Republic of Korea	Cross-sectional study	4	Good
Ottaviano *et al*.^34^	6	6 (100%) objective olfactory dysfunction	Padova, Italy	Case series	4	Fair
Paderno *et al*.^35^	508 (295 hospitalised + 213 home-quarantined)	44% in hospitalised group & 72% in home-quarantined group had subjective olfactory dysfunction	Brescia, Italy	Cross-sectional study	4	Good
Speth *et al*.^36^	103	61.2% subjective olfactory dysfunction	Aarau, Switzerland	Cross-sectional study	4	Good
Spinato *et al*.^37^	202	130 (64.36%) subjective mixed olfactory &/or taste dysfunction	Treviso, Italy	Cross-sectional study	4	Fair
Tostmann *et al*.^38^	79	37 (46.8%) subjective olfactory dysfunction	Nijmegen, Netherlands	Cross-sectional study	4	Fair
Trubiano *et al*.^39^	28	11 (39.3%) subjective mixed olfactory &/or taste dysfunction	Melbourne, Australia	Retrospective cohort study	4	Fair
Tsivgoulis *et al*.^40^	22	16 (72.7%) objective olfactory dysfunction	Athens, Greece	Cross-sectional study	4	Good
Vaira *et al*.^41^	345	256 (74.2%) subjective chemosensitive disorders, but 30.1% of 89 patients who did not report dysfunction proved objectively hyposmic	Sassari, Salerno, Milan & Bologna, Italy	Cross-sectional study	4	Good
Vaira *et al*.^42^	72	60 (83.33%) objective dysfunction; 44 (61.1%) subjective dysfunction	Sassari, Italy	Case series	4	Good
Vaira *et al*.^43^	33	25 (75.76%) had dysfunction on objective & self-administered test; 17 (51.52%) had subjective dysfunction	Sassari, Bologna & Salerno, Italy	Cross-sectional study	4	Fair
Wee *et al*.^44^	154	22.7% subjective mixed olfactory &/or taste dysfunction	Singapore	Cross-sectional study	4	Poor
Yan *et al*.^2^	59	40 (67.8%) subjective dysfunction	La Jolla, USA	Cross-sectional study	4	Good
Yan *et al*.^45^	128	75 (58.59%) subjective dysfunction	La Jolla, USA	Cross-sectional study	4	Good
Zayet *et al*.^46^	95	60 (63.2%) dysfunction	Trévenans, France	Retrospective cohort study	4	Good

Covid-19 = coronavirus disease 2019; NHI-SQAT = National Heart, Lung, and Blood Institute Study Quality Assessment Tools

Olfactory ability was assessed by: using validated questionnaires focused on smell dysfunction, in three studies; obtaining objective information on smell impairment through standardised chemosensitive tests with odorants, in five studies; and considering both methods, in five studies (Table 3).^18,20,24–26,29,32,34,37,40–43,47–59^ The remaining studies assessed olfactory ability through anamnestic data collection, simple surveys and/or structured, non-validated questionnaires (Table 4).^2,16,17,19,21–23,27,28,30,31,33,35,36,38,39,44–46^

**Table 3.** Summary of smell-related outcomes assessed via validated questionnaires and/or objective tests

Study authors	Patient age (years)	Setting	Olfactory evaluation(s)	Evaluation time point	Evaluation results	Olfactory dysfunction onset
Carignan *et al*.^18^	Median 57.1; IQR 41.2–64.5	Out-patients (except 3 Covid-19 patients admitted to hospital)	Adapted questions from Self-reported Mini Olfactory Questionnaire^47^	Within 72 hours’ (before or after) SARS-CoV-2 testing	Anosmia 69 (51.5%), dysgeusia 85 (63.4%)	3 (2.2%) reported anosmia & dysgeusia as presenting manifestations
Hornuss *et al*.^20^	Median 56 ± 16.9	In-patients	Self-report questionnaire, Burghart Sniffin’ Sticks smell test^48,49^	N/A	44% of anosmic & 50% of hyposmic patients on objective tests did not report smelling problems	N/A
Lechien *et al*.^24^	Average 36.9 ± 11.4; IQR 19–77	Non-ICU in-patients & infected healthcare workers across Europe	sQOD-NS^50^	Average of 9.2 ± 6.2 days after first symptoms onset	Anosmia 284, 73 hyposmia	Olfactory dysfunction appeared before (11.8%), after (65.4%) or at same time as appearance of general or ENT symptoms (22.8%)
Lechien *et al*.^25^	Average 39.50; IQR 12.10	161 (8%) in-patients & 1852 (92%) out-patients	Standardised online validated questionnaire NAHNES;^51^ a subset of patients had Burghart Sniffin’ Sticks smell test^48,49^	Mean (SD) time from end of disease to evaluation was 7.8 (6.8) days	Mean duration of olfactory dysfunction was 8.4 days (SD, 5.1)	Before other symptoms (15%), concomitant with other symptoms (25%) or after other symptoms (57%) (considering patients with smell dysfunction)
Lechien *et al*.^26^	Mean 41.7 ± 11.8	Out-patients	NAHNES,^51^ sQOD-NS,^50^ SNOT-22 (French version), Burghart Sniffin' Sticks smell test^48,49^	Mean duration of olfactory dysfunction at evaluation time was 17 ± 11 days for anosmic & 18 ± 11 days for hyposmic patients	Objective olfactory testing: 41 (47.7%) anosmic, 12 (14.0%) hyposmic	61.4% of patients described total loss of smell at disease onset
Li *et al*.^29^	Average 49 (range, 13–80)	Multicentre prospective cohort study	Smell identification testing using a T&T olfactometer based scoring system^52^ with odours generally familiar to Chinese population	N/A	Dysosmia of: garlic in 7 (5%), pineapple in 13 (9%), mint in 11 (8%) & ginger in 38 (26%)	Average from symptom onset of 62 days (range, 25–95)
Moein *et al*.^32^	Average 46.55 ± 12.17 (overall population)	In-patients in single hospital	UPSIT smell test,^53^ single question	Patients dismissible within 4 days	Anosmia in 15; microsmia was severe in 20, moderate in 16 & mild in 8	N/A
Ottaviano *et al*.^34^	N/A	N/A	Objective olfactory test ‘Le Nez Du Vin’ quick olfaction test,^54^ PROMs,^34^ SNOT-22,^55^ smell & taste VAS^55^	N/A	Alterations in smell & taste; nasal symptoms other than olfaction or taste were found to be irrelevant	N/A
Spinato *et al*.^37^	Median 56; IQR 45–67	Out-patients in single hospital	ARTIQ,^56^ SNOT-22^55^	Patients were asked if had experienced sudden onset of altered smell or taste in 2 weeks before swab	SNOT-22 grades: 5 very mild, 23 mild, 27 moderate, 27 severe, 48 as bad as it can be	Timing of altered sense of smell or taste onset in relation to other symptoms occurred before other symptoms in 24 (11.9%), at same time in 46 (22.8%) & after other symptoms in 54 (26.7%)
Tsivgoulis *et al*.^40^	Mean 55 ± 10	In-patients	SNOT-22,^55^ Q-SIT (Sensonics, Haddon Heights, NJ, USA)^57^	N/A	Microsomia in 15, anosmia in 1	N/A
Vaira A *et al*.^41^	Average 48.5 ± 12.8 (range, 23–88)	184 in-patients & 161 out-patients	CCCRC orthonasal olfaction test^58,59^ administered for hospitalised patients; test with 7 groups of odorants for home-quarantined patients	9.9 ± 5.8 (range, 1–28) days from positive swab; 14.8 ± 7.4 (range, 2–35) days from Covid-19 symptoms onset	Normal findings in 104 (30.1%); hyposmia was mild in 76 (22%), moderate in 59 (17.1%), severe in 45 (13%); anosmia in 61 (17.7%)	High frequencies of olfactory disorders throughout observation period, ranging between 77.4% (days 1–4) & 69.2% (days 25–35)
Vaira *et al*.^42^	Average 49.2 ± 13.7 (range, 26–90)	In-patients in single teaching hospital & infected healthcare workers	CCCRC orthonasal olfaction test,^58,59^ single questions	Average 19.3 ± 4.5 days from onset; 15.6 ± 4.3 days from positive swab. Prevalence over whole disease course	Hypogeusia was mild in 22, moderate in 33, severe in 3; ageusia in 2; olfactory dysfunction in 44	N/A
Vaira *et al*.^43^	Average 47.2 ± 10 (range, 26–64)	Out-patients in 3 hospitals	CCCRC orthonasal olfaction test,^58,59^ self-administered home test, single questions	Average 20.1 ± 3.9 days from onset; 17.5 ± 3.1 days from positive swab. Prevalence over whole disease course	Of 21 with chemosensory dysfunctions, 4 had hyposmia only, 4 had anosmia hyposmia only, & 13 reported olfactory & taste disorders	N/A

IQR = interquartile range; Covid-19 = coronavirus disease 2019; SARS-CoV-2 = severe acute respiratory syndrome coronavirus-2; N/A = not applicable; ICU = intensive care unit; sQOD-NS = short version of the Questionnaire of Olfactory Disorders – Negative Statements (a seven-item patient-reported outcome questionnaire including social, eating, annoyance and anxiety questions; NAHNES = National Health and Nutrition Examination Survey; SD = standard deviation; UPSIT = University of Pennsylvania Smell Identification Test; PROM = patient-reported outcome measures; SNOT-22 = Sino-Nasal Outcome Test-22; VAS = visual analogue scale; ARTIQ = Acute Respiratory Tract Infection Questionnaire; Q-SIT = Quick Smell Identification Test; CCCRC = Connecticut Chemosensory Clinical Research Center

**Table 4.** Summary of smell-related outcomes assessed via anamnestic data collection, simple surveys and/or non-validated questionnaires

Study authors	Patient age (years)	Setting	Olfactory evaluation(s)	Evaluation time point	Evaluation results	Olfactory dysfunction onset
Aggarwal *et al*.^16^	Mean 65.5	In-patients in single hospital	Electronic medical record database	N/A	Anosmia 3 (19%), dysgeusia 3 (19%)	N/A
Beltrán-Corbellini *et al*.^17^	Average 61.6 ± 17.4	In-patients in multiple (*n* = 2) tertiary care hospitals	Non-validated questionnaire	N/A	Anosmia 14 (17.7%), ageusia 14 (17.7%)	22 (27.8%) had acute onset of olfactory &/or taste dysfunction; first symptom in 11 (13.9%)
Giacomelli *et al*.^19^	Median 60; IQR 50–74	In-patients in single tertiary care hospitals	Non-validated questionnaire (single question)	Median of 15 days after first symptoms onset	Anosmia 7 (11.9%), hyposmia 7 (11.9%)	N/A
Kai Chua *et al*.^21^	N/A	Patients referred to single tertiary care hospital with acute respiratory symptoms	Non-validated questionnaire	N/A	Hyposmia 3 (9.6%), anosmia 4 (12.9%)	N/A
Kim *et al*.^22^	Median 26; IQR 22–47	Community designated for isolation of Covid-19 patients	Non-validated questionnaire survey	N/A	Of 68 individuals with hyposmia, 61 had accompanying symptoms such as hypogeusia, nasal congestion or rhinorrhoea	N/A
Klopfenstein *et al*.^23^	Average 47 ± 16 (for patients with olfactory disorders only)	In-patients & out-patients in single hospital	Non-validated questionnaire (single question)	Prevalence over whole disease course	Anosmia 54 (47.4%)	Olfactory dysfunction was never first symptom; onset 4.4 days after
Lechien *et al*.^27^	Mean 39.17 ± 12.09	In-patients & out-patients	Non-validated standardised questionnaire	N/A	Loss of smell 70.2%, nasal obstruction 67.8%, rhinorrhoea 60.1%, gustatory dysfunction 54.2%	Loss of smell persisted at least 7 days after disease in 37.5% of cured patients. Mean duration of olfaction dysfunction was 8.41 ± 5.05 days
Lee *et al*.^28^	Average 36.5 (range, 24.5–54.0)	Out-patients awaiting hospitalisation or facility isolation	Single question	Early stage of disease	Anosmia & ageusia in 254 of 488 (52.0%), ageusia only in 99 (20.3%), anosmia only in 135 (27.7%)	Early stage of Covid-19
Luers *et al*.^30^	Average 38 ± 13 (range, 21–87) for overall population	Out-patients in single teaching hospital	Single question	Average of 13 ± 3 days after first symptoms; 7 ± 1 after positive swab	Olfactory dysfunction 53 (73.61%)	N/A
Menni *et al*.^31^	Average of 41.25 ± 12.18 in UK cohort & 44.65 ± 14.31 in US cohort	Out-patients	Self-reported symptoms – ‘COVID RADAR Symptom Tracker app’ (question on symptoms)	N/A	64.8% in UK & 67.5% in USA had subjective olfactory &/or taste dysfunction	N/A
Noh *et al*.^33^	Mean 38.0	Patients in residential treatment centre	Single questions	N/A	52 (26.1%) anosmia, 45 (22.6%) ageusia	Duration of anosmia ranged 2–28 days (median, 7 days)
Paderno *et al*.^35^	Mean 55 ± 15	In-patients & out-patients	Non-validated, survey-based questionnaire focusing on olfactory & gustatory dysfunctions	Mean lag time between swab & survey was 11 ± 8 days	Subjective olfactory dysfunction in 44% in hospitalised group & in 72% in home-quarantined group	Mean lag time between symptom onset & survey was 18 ± 7 days
Speth *et al*.^36^	Mean 46.8 ± 15.9	In-patients & out-patients	Non-validated standardised questionnaire	N/A	14.6% hyposmia, 46.6% anosmia	Olfactory dysfunction was experienced on 1st day of disease by 8.7%
Tostmann *et al*.^38^	N/A	Healthcare workers in single teaching hospital	Non-validated questionnaire	N/A	37 (46.8%) subjective olfactory dysfunction	N/A
Trubiano *et al*.^39^	Median 55 (IQR 46, 63.5)	Patients previously assessed in single hospital	Hospital dataset	N/A	7 (25%) anosmia (with or without ageusia); 7 (25%) ageusia (with or without anosmia); 3 (10.7%) anosmia & ageusia	N/A
Wee *et al*.^44^	N/A	In-patients & out-patients in single hospital	Non-validated questionnaire including self-reported olfactory & gustatory dysfunctions	N/A		N/A
Yan *et al*.^2^	N/A	Out-patients & in-patients in single hospital	Single question	Prevalence over whole disease course	Olfactory dysfunction in 40	22% reported anosmia as first symptom
Yan *et al*.^45^	Median 53.5 & IQR 40–65 for in-patients; median 43 & IQR 34–54 for out-patients	Out-patients & in-patients in single hospital	Single question	Prevalence over whole disease course	olfactory dysfunction in 75	N/A
Zayet *et al*.^46^	Mean 39.8 ± 12.2 (range, 18–73)	Out-patient in single hospital	Non-validated standardised questionnaire	N/A	Dysgeusia & anosmia in 52 (54.7%), dysgeusia &/or anosmia in 70 (73.7%)	N/A

N/A = not applicable; IQR = interquartile range; Covid-19 = coronavirus disease 2019

## Discussion

Our review confirmed that olfactory disorders represent an important clinical feature in individuals affected by Covid-19, with a prevalence ranging from 11 per cent to 100 per cent of included patients, although there was heterogeneity in terms of assessment tools and population selection criteria.

The reported data show that smell dysfunction was, overall, more prevalent in patients investigated with validated questionnaires and/or tests with odorants (Table 3), compared to individuals evaluated using anamnestic data, simple surveys and/or non-validated questionnaires. This is in agreement with the findings of Moein *et al*.^32^ and further studies,^60^ which indicate that self-reported evaluations of olfactory loss are not in line with the more reliable outcomes of standardised tests. There are exceptions to this general trend, however, as highlighted by the papers of Lechien *et al*.^26^ and Li *et al*.,^29^ but in these latter manuscripts there are some possible biases that may affect the data.

The variations in reported outcomes may be a result of the different methods of evaluation; however, the variations might also be because of other important factors. Primarily, non-validated tests are only focused on smell disorders of new onset and do not investigate the presence of olfactory dysfunction prior to Covid-19. In contrast, a validated questionnaire and/or objective olfactory test allows greater accuracy regarding the real prevalence of olfactory disorders, the exact timing of onset and their characteristics.

Furthermore, our analysis does not suggest any significant differences in terms of the age or gender of the enrolled subjects, although younger patients often seem to show a greater prevalence of smell disorders than older ones. These data seem difficult to understand until we consider that the elderly population has a higher prevalence of smell disorders overall. In the context of Covid-19, younger patients are more likely to have a new onset of olfactory dysfunction (more evident with a non-validated questionnaire analysis), and frequently have less severe respiratory symptoms, resulting in more susceptibility to olfactory problems. Therefore, we believe that age should be considered as a possible bias, at least regarding the elderly population, given that the estimated prevalence of smell impairment in the general population aged above 80 years ranges between 43.1 per cent and 84.9 per cent.^61^

Regarding the hospital setting, our review highlighted a lower prevalence of smell disorders in hospitalised patients compared with home-quarantined patients. Two studies focused specifically on this comparison,^2,35^ emphasising a greater prevalence of the disorder in individuals with low-to-mild disease compared to those who needed hospital treatment. Once again, this difference could be related to greater attention devoted to olfactory impairment in patients in an overall better health condition.

Another relevant source of heterogeneity is linked to the different timings of smell evaluation with respect to the onset of symptoms. According to our data, smell dysfunction seems to occur mostly in early stages of the disease, and tends to decrease or resolve within the two weeks following virologic healing in the majority of the patients; therefore, all evaluations that take place during an advanced or unspecified disease stage could underestimate olfactory dysfunction.

Finally, we should consider that the large prevalence of smell disorders apparently became evident only when the SARS-CoV-2 infection hit Europe. In the first studies performed in China and Singapore, patients were frequently unaware of olfactory dysfunction.^62–64^ It is striking that more than half of the reviewed studies were carried out in European countries. This could be related to a higher prevalence of Covid-19 associated smell disorders in Caucasian people, although other factors should be taken into account. A possible bias could be presented by the fact that some scientific reports are written in original Chinese language and are difficult to access. In addition, we should bear in mind that – with the exception of China – the scholarly production on Covid-19 and olfactory dysfunction follows the outbreak spread, which is already peaking in Europe and Western Asia, has flourished in North America and is in an earlier stage in South America.

While these data confirm what has already been included in earlier reviews, our paper is able to present a somewhat later analysis of the issue of smell impairment in Covid-19. It discusses more complete and well-defined data than other previously published papers, and includes a significantly greater number of patients.

Nevertheless, many problems need to be addressed to allow a holistic evaluation of smell impairments in Covid-19 patients. In order to allow further and stronger meta-analytic papers, smell assessment tools should converge into validated questionnaires and odorant tests. In addition, important reported biases (e.g. age, hospital setting and patients’ overall condition) should be appropriately addressed in the context of well-designed future prospective studies.

## Conclusion

In the wake of the relevance of olfactory dysfunction in individuals with Covid-19, we believe that olfactory assessment is essential in every patient with a new diagnosis of SARS-CoV-2 infection in the early stage. Furthermore, we think that smell disorders of new onset should be considered a possible symptom for suspected SARS-CoV-2 infection. Our study suggests the need for a clinical standardised evaluation carried out using structured questionnaires and, if possible, tests with olfactory substances. Finally, ENT assessment in Covid-19 patients should be routinely proposed to ensure the correct evaluation of chemosensitive disorders and the possible need for therapeutic strategies.
